# Cortical morphology in patients with transient global amnesia

**DOI:** 10.1002/brb3.872

**Published:** 2017-11-19

**Authors:** Hyung Chan Kim, Byung In Lee, Sung Eun Kim, Kang Min Park

**Affiliations:** ^1^ Department of Neurology Haeundae Paik Hospital Inje University College of Medicine Busan Korea

**Keywords:** amnesia, cortical thickness, magnetic resonance imaging, volume

## Abstract

**Objective:**

This study evaluated alterations in cortical morphology in patients with transient global amnesia (TGA).

**Materials and Methods:**

Diagnoses of TGA occurred at our hospital. Evaluation involved a structured interview to obtain clinical information and a brain magnetic resonance imaging scan. We analyzed whole‐brain T1‐weighted MRI data using FreeSurfer 5.1. Measures of cortical morphology, such as thickness, surface area, volume, and curvature were compared between patients with TGA and healthy controls. We also quantified the correlations between clinical variables and each measure of abnormal cortical morphology.

**Results:**

Seventy patients met the inclusion criteria. Compared to healthy controls, patients with TGA had significant alterations in cortical thickness in several regions of bilateral hemisphere. Moreover, several regions of cortical volumes in left hemisphere were significantly different between patients with TGA and healthy controls. In addition, there were significant correlations between the durations of episodes and cortical thickness, especially in the parietal cortex. However, there were no differences between groups in other measures of cortical morphology, including surface area and curvatures.

**Conclusions:**

We observed significant alterations in cortical morphology in patients with TGA; these alterations are implicated in the pathogenesis of TGA.

## 1 INTRODUCTION

Transient global amnesia (TGA) is characterized by sudden onset of transient memory impairment with anterograde and partial retrograde amnesia, which lasts for several hours before resolving completely (Bartsch & Deuschl, 2010). Although TGA is a well‐documented clinical condition, the pathogenesis of TGA remains unclear. Several hypotheses, including arterial transient ischemic attacks (TIA), epileptic seizures, transient mesial temporal ischemia induced by venous congestion, and migraine and its underlying cortical spreading depression have been postulated (Bartsch & Deuschl, 2010). However, because the thromboembolic risk of patients with TGA is much lower compared to TIA, it is unlikely that TGA is a form of TIA (Mangla, Navi, Layton, & Kamel, 2014). Furthermore, although patients with TGA have a higher prevalence of migraine than control participants, there is no direct evidence for cortical depression in patients with TGA (Lin et al., 2014). The low rate of TGA recurrence is not explained by seizures, and electroencephalography (EEG) does not demonstrate epileptiform abnormalities (Jacome, 1989).

Neuroimaging techniques have historically failed to elucidate structural abnormalities in patients with TGA. However, some recent evidence suggests that structural abnormalities are associated with TGA. Several cases have been reported, which suggest that brain structural abnormalities such as acute infarction of the cingulate gyrus and fornix may induce TGA (Gallardo‐Tur, Romero‐Godoy, de la Cruz Cosme, & Arboix, 2014; Gupta, Kantor, Tung, Zhang, & Albers, 2014). Recently, studies using diffusion‐weighted imaging MRI have consistently revealed that the acute stage of TGA is associated with precise focal lesions in hippocampal CA1 (Bartsch et al., 2006; Kim et al., 2014). Moreover, a previous study demonstrated that the incidence of hippocampal cavities is 100% in patients with TGA; cavities were considerably larger than standard cavities and were often bilateral. Hippocampal cavities occurred in only 31% of control participants (Nakada, Kwee, Fujii, & Knight, 2005). Although the exact mechanisms have not been elucidated, these focal lesions in the hippocampus and subsequent perturbation of the hippocampal‐neocortical memory system may be critical to the pathogenesis of TGA (Bartsch & Butler, 2013).

We recently used voxel‐based morphometry (VBM) to demonstrate that patients with TGA have significant gray matter volume reductions in the hippocampus, amygdala, cingulum, and cerebellum (Park et al., 2015). However, previous studies have not investigated cortical morphology in patients with TGA. Cortical volume is the product of surface area and thickness, and measures of cortical volume therefore combine cortical surface area and cortical thickness. Neurons within the cerebral cortex are organized into ontogenetic columns that run perpendicular to the surface of the brain (Panizzon et al., 2009). The size of the cortical surface area is determined by the number of columns, whereas cortical thickness is determined by the number of cells within a column (Rakic, 1988). Recent research has demonstrated that cortical surface area and cortical thickness are independent and result from distinct neuro‐biologic and genetic processes; these processes may contribute to the pathogenesis of neurological conditions such as TGA (Im et al., 2008; Panizzon et al., 2009; Rakic, 1988; Winkler et al., 2010). Thus, studies assessing cortical volume may be confounded by underlying brain structure. Therefore, measurements of surface area and cortical thickness should be evaluated separately. Moreover, cortical anatomy, which is structured as a corrugated two‐dimensional sheet of tissue, can be accurately represented by surface‐based analyses (SBA), which provide superior visualization compared to VBM (Van Essen, Drury, Joshi, & Miller, 1998).

We evaluated cortical morphology in patients with TGA, using SBA, with the aim to elucidate the mechanisms of TGA pathogenesis. In addition, we quantified the correlations between clinical variables and each measure of abnormal cortical morphology.

## 2 MATERIALS AND METHODS

### 2.1 Patients

This study was conducted with the approval of the Institutional Review Board at Inje University College of Medicine, conformed to the tenets of the Declaration of Helsinki, and was conducted at a single tertiary hospital. We prospectively enrolled 70 patients with TGA, which was diagnosed according to Hodges and Warlow's criteria, as follows (Hodges & Warlow, 1990): (1) episodes should be witnessed and the information collected from an observer who was present for a significant period during the episode, (2) presence of anterograde amnesia during the episode, (3) absence of conscious disturbances and no loss of personal identity, (4) cognitive deficits should be limited to amnesia, (5) absence of accompanying focal neurological disturbances during the episode, (6) epileptic features must be absent, (7) resolution of the episode within 24 hrs, and (8) exclusion of patients with recent trauma or with seizures. Patients were excluded if they met the following criteria: (1) visual structural lesions on brain MRI, (2) a history of neurological or psychiatric disease, due to the potential influence on cortical morphology, or (3) a history of drug abuse, which could interfere with cognitive performance. All patients underwent a detailed interview to obtain the following data: vascular risk factors (diabetes, hypertension, and dyslipidemia) and characteristics of the episode (number of episodes, duration, and precipitating event). Some of the patients were also included in the previous study (Park et al., 2015).

### 2.2 Healthy controls

The control group consisted of 35 age‐ and gender‐matched healthy participants. The mean age was 57.6 ± 11.4 years. Of the 35 healthy controls, 14 participants (40%) were men and 21 participants (60%) were women. All participants had normal neurological examinations and no history of any diseases. All healthy controls had normal MRIs on visual inspection.

### 2.3 MRI data acquisition, processing, and statistical analyses with FreeSurfer

The processes of MRI data acquisition, processing, and analyses using FreeSurfer were previously described (Park et al., 2016). Briefly, all scans were performed using a 3.0 T MRI scanner, and all participants underwent conventional brain MRI protocols, including collection of T1‐ and T2‐weighted images. Sagittal‐oriented high‐resolution contiguous 3D T1‐weighted images were also obtained.

The processing stream with FreeSurfer consisted of several stages, as follows: volume registration with the Talairach atlas, bias field correction, initial volumetric labeling, nonlinear alignment to the Talairach space, and final volume labeling. The differences in cortical morphology, including thickness, surface area, volume, and curvature were investigated between the patients with TGA and healthy controls. Measurements of cortical morphology were analyzed with independent samples *t* test using the Qdec application. Statistical significance was defined as *p *<* *.05. Multiple comparisons with family‐wise error were conducted; age and gender were included as covariates. We quantified the correlations between clinical variables and each measure of abnormal cortical morphology using Spearman's rank correlations without multiple corrections.

Categorical clinical variables are presented as frequencies and percentages. Numerical variables with normal distributions are presented as means ± standard deviations, and numerical variables without normal distributions are presented as medians with 95% confidence intervals and ranges. Statistical significance was indicated by *p*‐values <.05. All statistical tests were performed using MedCalc^®^ (software version 13, Ostend, Belgium).

## 3 RESULTS

### 3.1 Demographic and clinical characteristics of patients

Seventy patients met the inclusion criteria. Twenty‐five patients (36%) were men, and 45 patients (64%) were women. The mean age was 60.8 ± 8.1 years. The current TGA episode was the first such event in 65 patients, and five patients reported previous episodes of TGA (two patients with two episodes, two patients with three episodes, and one patient with seven episodes). The median duration of amnesia was 6 hrs (95% CI 4.0–7.0 hrs, range 1–18 hrs). Twenty‐five (36%) of the 70 patients had a history of vascular risk factors (19 patients with hypertension, four patients with dyslipidemia, and two patients with diabetes). Fifty patients (71%) had a history of precipitating events (35 patients with emotional stress, eight patients with physical effort, and seven patients with water contact/ temperature change).

### 3.2 Measures of cortical morphology: thickness, surface area, volume, and curvature

**Figure 1:**
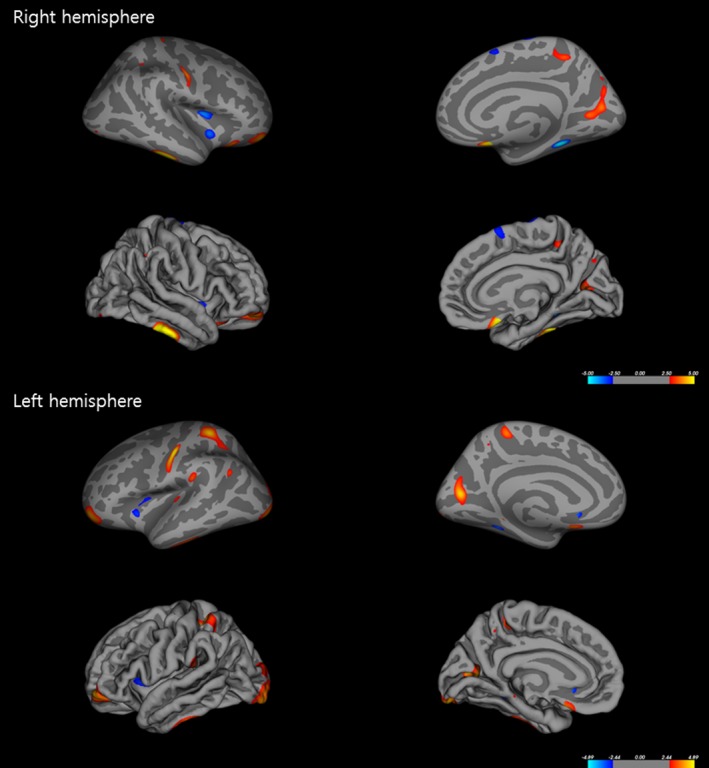
Alterations in cortical thickness in patients with transient global amnesia (TGA). Regions with significantly increased cortical thickness are indicated in red, whereas blue represents regions with decreased cortical thickness in patients with TGA compared to healthy controls (multiple corrections with *p *<* *.05)

**Figure 2:**
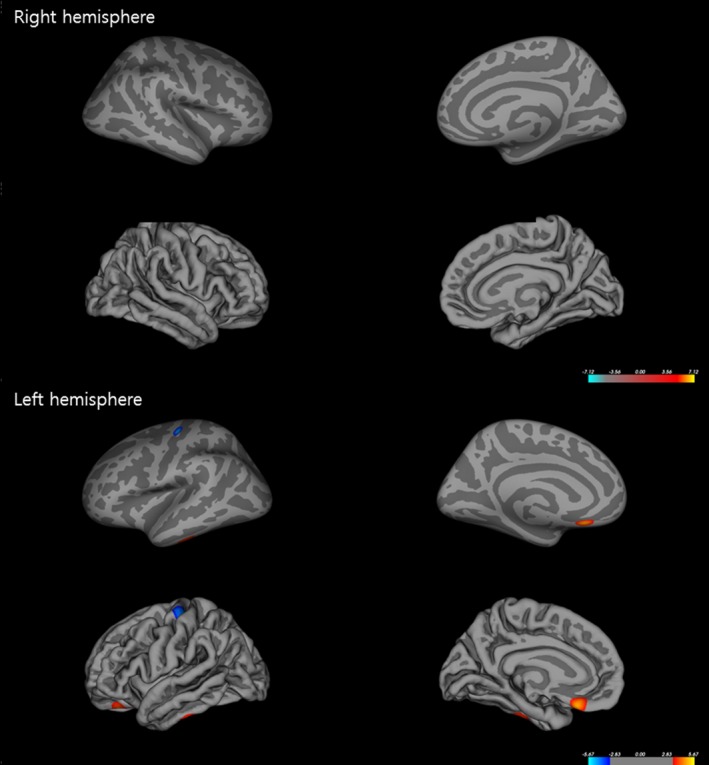
Alterations in cortical volume in patients with transient global amnesia (TGA). Regions of significantly increased cortical volume are indicated in red, whereas blue represents regions with decreased cortical thickness in patients with TGA compared to healthy controls (multiple corrections with *p *<* *.05)

Measures of cortical thickness revealed significant cortical alterations in several regions in patients with TGA compared to healthy controls (Figure 1). In patients with TGA, decreased cortical thickness was detected in the right hemisphere, including in the fusiform, insula, precentral, and superior frontal cortex, and in the left hemisphere, including in the fusiform, anterior cingulate, and insular cortex. In contrast, cortical thickness increased in the right hemisphere, including inferior temporal, medial and lateral orbitofrontal, middle frontal, postcentral, pericalcarine, precuneus, superior parietal, and lateral occipital cortex, as well as in the left hemisphere, including postcentral, pericalcarine, superior and inferior parietal, lateral occipital, middle frontal, inferior and transverse temporal, paracentral, medial orbitofrontal, supramarginal, parahippocampal, and precuneus cortex of patients with TGA compared to healthy controls. In addition, the measures of cortical volume revealed significant cortical alterations in several regions in patients with TGA (Figure 2). There was decreased cortical volume in the left hemisphere, including precentral cortex, whereas increased cortical volume was observed in the left hemisphere, including medial and lateral orbital frontal and inferior temporal cortex. However, right hemisphere cortical volumes were not significantly different between patients with TGA and healthy controls. There were also no differences between groups in other measures of cortical morphology, including surface area and curvatures.

### 3.3 Correlations between measures of abnormal cortical morphology and clinical variables

Cortical thicknesses generally correlated with the durations of episodes (Table 1). In particular, the cortical thickness of the precuneus, superior parietal, and lateral occipital cortex in the right hemisphere, and the fusiform, postcentral, superior and inferior parietal, paracentral, and precuneus in the left hemisphere, were significantly correlated with the durations of episodes. However, there were no correlations between measures of abnormal cortical volume and the durations of episodes in patients with TGA. In addition, the number of episodes was not correlated with abnormal cortical morphologies, including thickness and volume.

**Table 1:** Correlations between abnormal cortical thickness/ volume and number/ duration of episodes in patients with transient global amnesia (TGA)

Cortical morphology	Number of episodes		Duration of episodes	
	*r*	*p*	*r*	*p*
Thickness in right hemisphere				
Fusiform	.045	.711	.142	.268
Insula	−.063	.605	.016	.901
Precentral	.064	.601	.222	.080
Superior frontal	−.015	.902	.146	.254
Inferior temporal	.019	.878	−.074	.564
Medial orbitofrontal	−.054	.656	.139	.277
Lateral orbitofrontal	−.001	.991	−.052	.683
Middle frontal	.034	.782	.226	.075
Postcentral	−.039	.748	.232	.067
Pericalcarine	−.048	.694	.223	.080
Precuneus	.011	.927	.449	<.001^a^
Superior parietal	−.028	.816	.460	<.001^a^
Lateral occipital	−.065	.595	.280	.026^a^
Thickness in left hemisphere				
Fusiform	−.049	.690	.260	.040^a^
Anterior cingulate	−.041	.739	−.111	.385
Insula	−.066	.585	.069	.590
Postcentral	−.106	.381	.302	.016^a^
Pericalcarine	.006	.961	.218	.087
Superior parietal	.014	.910	.478	<.001^a^
Inferior parietal	.011	.928	.345	.006^a^
Lateral occipital	−.093	.445	.149	.244
Middle frontal	.053	.665	.166	.192
Inferior temporal	.024	.843	.195	.126
Transverse temporal	−.144	.233	.162	.205
Paracentral	.099	.414	.270	.033^a^
Medial orbitofrontal	.034	.783	.009	.942
Supramarginal	.123	.309	.233	.066
Parahippocampal	.009	.938	.066	.610
Precuneus	.011	.927	.296	.018^a^
Volume in left hemisphere				
Precentral	.068	.577	.116	.3635
Medial orbitofrontal	−.207	.085	−.043	.7388
Lateral orbitofrontal	−.147	.223	.072	.5753
Inferior temporal	.057	.640	−.074	.5668

a *p *<* *.05.

## 4 DISCUSSION

This is the first study to investigate alterations in cortical morphology in patients with TGA, using SBA. We enrolled 70 patients, which is a relatively large sample. Our main finding is that patients with TGA have widespread alterations in cortical morphology, including cortical thickness and volume, compared to healthy controls. This finding is consistent with previous single‐photon emission computed tomography (SPECT) (Jang et al., 2015), positron emission tomography (PET) (Baron et al., 1994), and resting state functional MRI (rs‐fMRI) (Peer et al., 2014) studies, which have also demonstrated changes in network regions in addition to the hippocampus in patients with TGA. This study demonstrates that abnormal cortical thickness, especially in the parietal cortex, is correlated with episode durations.

The regions of cortical alterations in this study were different from those in our previous study (Park et al., 2015). The differences probably originated from the differences in analyzing methods (VBM in the previous study vs. SBA in this study). VBM exhibits limited accuracy in the measurement of cortical morphology, especially in brain regions where fine anatomic details are often obscured by a partial volume effect due to a corrugated two‐dimensional sheet of tissue (Im et al., 2008; Panizzon et al., 2009; Rakic, 1988; Van Essen et al., 1998; Winkler et al., 2010). SBA reflects more accurate cortical geometry and overcome partial volume effect. Surface‐based analyses can make it possible to measure cortical thickness in millimeters with more precise measurement in deep sulci as a cortical sheet. In addition, cortical shape and folding pattern can be analyzed, which is not possible in VBM (Im et al., 2008; Panizzon et al., 2009; Rakic, 1988; Van Essen et al., 1998; Winkler et al., 2010).

This study revealed widespread alterations in cortical thickness in patients with TGA, without alterations to the cortical surface area. This finding indicates that alterations in cortical thickness, rather than altered cortical surface areas, are the primary initiator of alterations in cortical volume in patients with TGA. These distinct alteration patterns reflect independence as biomarkers for different neuro‐pathologic mechanisms. In healthy human brains, large cortical volumes are accompanied by large increases in cortical surface areas and markedly smaller increases in cortical thickness. The alterations in cortical thickness observed in this study were not caused by between‐participant differences in cortical volumes (Im et al., 2008). Thus, although the underlying pathogenesis of cortical thickness alterations in patients with TGA is not yet fully understood, these changes may result from neuronal injury in the cortex, which may decrease the number of cells within columns (Labate et al., 2011). Our findings of altered cortical thickness are consistent with previous reports on other neurological disorders such as epilepsy (Park et al., 2016).

Although we observed bilateral alterations in cortical morphology in patients with TGA, there were asymmetric patterns of alterations, including for cortical thickness, with predominance in the left hemisphere. In addition, alterations in cortical volume were only evident in the left hemisphere. These findings are compatible with our previous study using VBM, which also found volume reductions in the left hippocampus only. This left lateralization is consistent with other previous studies using EEG and SPECT (Chung et al., 2009; Jang et al., 2015; Kwon et al., 2014). Kim et al. analyzed EEG abnormalities in patients with TGA; epileptiform discharge, or focal slowing, was detected in the left hemisphere only (54.3%) or in both hemispheres (28.6%), but not in the right hemisphere only (0%) (Kwon et al., 2014). The same research group also found, via SPECT analysis, that patients with TGA had significantly decreased cerebral perfusion in the left hemisphere only (Jang et al., 2015). They proposed that the effect of left cerebral hypo‐perfusion in amnestic events in patients with TGA may be greater than for the right hemisphere (Jang et al., 2015). Another SPECT study reported significantly decreased regional cerebral blood flow, with more prominent left hemisphere reductions in the superior temporal, precentral, and postcentral gyri in patients with TGA (Chung et al., 2009). These researchers suggested that lateralized abnormalities in brain functioning are an important component of the pathophysiology of TGA (Chung et al., 2009). Seizures that originate in the dominant hemisphere are associated with longer postictal recovery, and may also produce more excitotoxic damage. Furthermore, brain tissue in the dominant hemisphere may be more susceptible to damage than tissue in the nondominant hemisphere (Lu et al., 2013; Theodore, 2010). The left hemisphere is dominant in most participants, particularly since most participants were right‐handed in this study.

We found a correlation between the durations of episodes and abnormal cortical thickness, which was primarily restricted to the parietal cortex, including superior and inferior parietal, post central, para central, and precuneus cortex. These findings support a contribution of compromised parietal cortex to TGA, and may indicate a pathologic process responsible for TGA. Previous studies using SPECT and EEG have reported that cortical regions including parietal cortex exhibit altered neuronal activities, which may contribute to the pathogenesis of TGA (Jang et al., 2015; Park et al., 2016). There are two separate large‐scale cortical memory networks: the anterior temporal system and the posterior medial system (Ranganath & Ritchey, 2012). The anterior temporal system includes the perirhinal cortex, temporopolar cortex, lateral orbitofrontal cortex, and amygdala. This system processes familiarity‐based recognition memory, emotional processing, and social cognition; the posterior medial system, which includes the parahippocampal cortex, retrosplenial cortex, anterior thalamic nuclei, mammillary bodies, parasubiculum, and parietal cortex, is involved in episodic memory (Ranganath & Ritchey, 2012). The core symptom of TGA is substantial episodic memory impairment (Guillery‐Girard et al., 2004); therefore, posterior medial system disruption may contribute to TGA. This possibility is consistent with the present finding that cortical morphology is altered in the posterior medial system.

We found that patients with TGA had altered cortical morphology in most default mode network (DMN) regions. The DMN includes the prefrontal, cingulate, angular, and precuneus cortex, and it is broadly associated with internally directed cognition such as episodic memory, theory of mind, and self‐evaluation (Jeong, Chung, & Kim, 2015; Sestieri, Corbetta, Romani, & Shulman, 2011). Default mode network regions are typically deactivated during cognitive tasks and memory encoding tasks (Jeong et al., 2015). A previous rs‐fMRI study revealed that DMN regions are associated with greater activity during “remembering” than during “knowing” responses by healthy participants. This finding is consistent with a role of the DMN in self‐referential processing (Kim, 2010). Another study focused on memory retrieval in relation to the parietal regions inside and outside the DMN. During memory retrieval, DMN activity peaked earlier than in non‐DMN regions. The authors suggested that DMN parietal regions directly support memory retrieval, whereas non‐DMN parietal regions are primarily involved in post‐retrieval processes such as memory‐based decision‐making (Sestieri et al., 2011). This suggestion is consistent with the concept that hippocampal atrophy alone cannot induce episodic memory impairment in dementia, and that large‐scale neural networks such as the DMN have a role in episodic memory processing (Bartsch et al., 2010; Jeong et al., 2015; La Joie et al., 2014). A recent study examining DMN connectivity during aging also revealed that reduced DMN connectivity is associated with deficits in memory performance (Ward et al., 2015). Thus, cortical involvement of DMN regions is associated with impaired episodic memory in patients with TGA.

These are several limitations to this study. First, we did not evaluate or analyze detailed neuropsychological assessments. Second, it can be difficult to reveal a causal relationship between changes in cortical morphology and TGA development from this study. To identify the causal relationship between them, brain MRI should be conducted prior to events of TGA. Third, we did multiple correction analysis in investigating the differences in cortical morphology between the patients with TGA and healthy controls. However, multiple corrections were not conducted in the correlation analysis between the measures of abnormal cortical morphology and clinical variables. Therefore, it requires further validations in future studies.

## 5 CONCLUSION

To the best of our knowledge, this is the first study to investigate cortical morphology in patients with TGA. We describe evidence for significant widespread alterations in cortical morphology, which may be implicated in the pathogenesis of TGA. Future studies including neuropsychological and functional assessments should be conducted to expand on the present findings.

## CONFLICT OF INTEREST

None.
